# Supplementary material for: Predictive goal coding by dentate gyrus somatostatin-expressing interneurons in male mice
Source: Nat Commun. 2025 Jun 25;16:5382. doi: 10.1038/s41467-025-60841-y (PMC12198415; doi:10.1038/s41467-025-60841-y)
Supplement: Supplementary file 1 — Supplementary Information [file 41467_2025_60841_MOESM1_ESM.pdf]

Extended Data:

Predictive goal coding by dentate gyrus somatostatin-expressing interneurons in male mice

Mei Yuan[1,2], Aurore Cazala[1], Sven Goedeke[2,3], Christian Leibold[2,3], Jonas-Frederic Sauer[1,4], Marlene Bartos[1*]

1 Institute for Physiology I, University of Freiburg, Medical Faculty, 79104 Freiburg, Germany

2 Biological Faculty, University of Freiburg, 79104 Freiburg, Germany

3 Bernstein Center Freiburg, University of Freiburg

4 Institute of Physiology, Center for Integrative Physiology and Molecular Medicine, Medical Faculty, Saarland University, 66421 Homburg, Germany

* Correspondence: marlene.bartos@physiologie.uni-freiburg.de

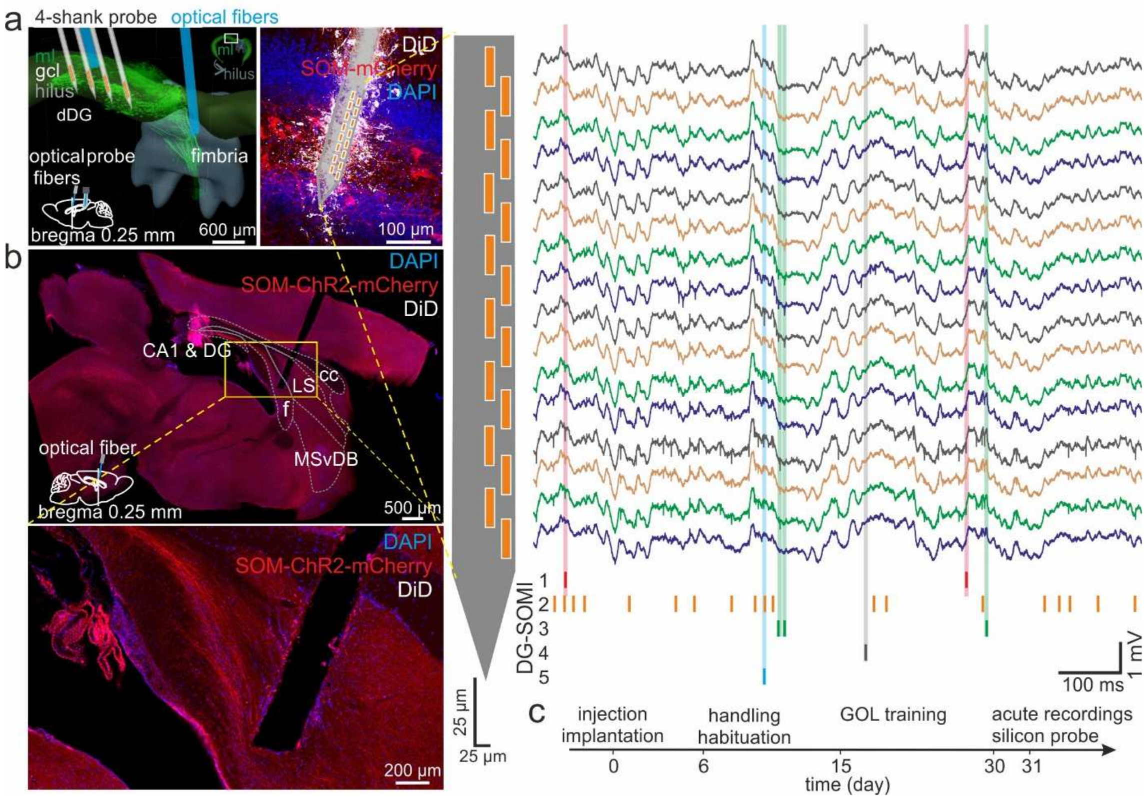

Supplementary Fig. 1. Extracellular local field potential recordings and behavioral timeline.

a Representative unfiltered recordings from one example shank with 16 recording sites. Upper left, schematic illustration of the recording probe implanted with the optical fiber in the dorsal DG and a second optical fiber positioned above the fimbria. SOM-expressing cells project with their axons via the fimbria to the medial septum (adapted from [21]). Right, unfiltered LFP recordings are shown from one example shank located in the hilus (upper right). DiD labeling of cell membranes confirmed the location of the probe in the hilar area. Color code of LFP traces represents the individual recording sites. Bottom, raster plots of 5 simultaneously recorded SOMIs.

b A sagittal section of the brain confirmed the implanted optical fiber position above the fimbria (f).

c Schematic of the experimental timeline. Animals were recorded for two consecutive days (day 30 and 31) with one recording session per hemisphere per day. Abbreviations: cc corpus callosum; dDG dorsal DG; f fimbria; gcl granule cell layer; LS lateral septum; ml molecular layer; MSvDB medial septum and vertical limb of the diagonal band of Broca.

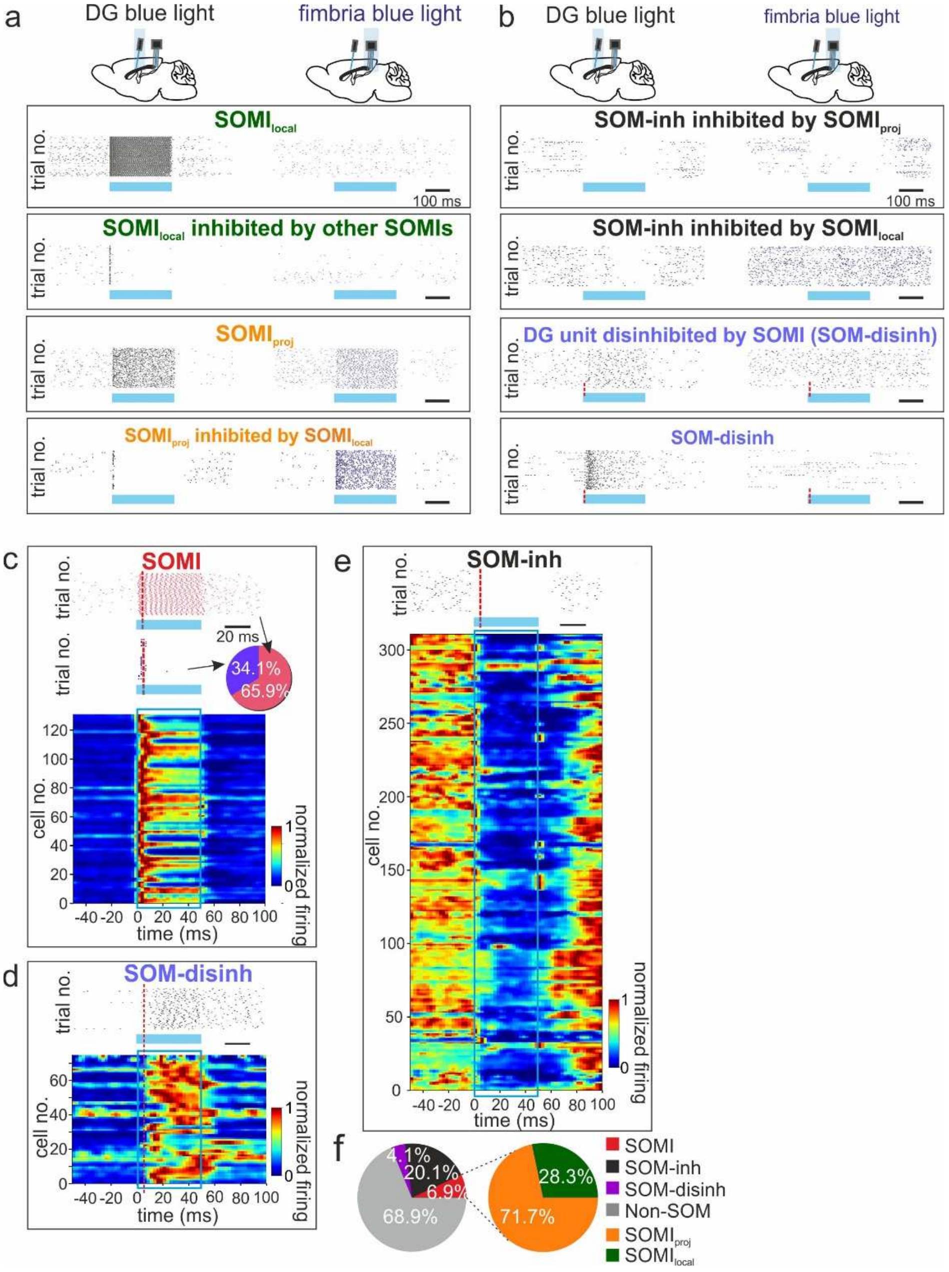

Supplementary Fig. 2. Optogenetic identification of SOMI subtypes and units modulated by SOMIs.

a-b To differentiate between DG-SOMIs projecting to the medial septum and the diagonal band of Broca (SOM_{proj}) and those with axons located within the hilus (SOM_{local}), blue light was delivered to the dDG (left row) and to the fimbria (right row). Fimbria-stimulation evoked action potentials in long-range projecting axonal fibers of SOMIs expressing ChR2, which retrogradely propagated to the recording side in the hilar dDG. SOMIs responding to fimbria stimulation were classified as putative $SOMI_{proj}$ while the ones responding only to dDG light-mediated stimulation as $SOMI_{local}$. Note that light delivery to the fimbria or to the DG caused inhibition of some DG units very likely by presynaptic $SOMI_{local}$ or by $SOMI_{proj}$ (SOM-inh). Although DG SOMIs do not show adaptation of high-frequent trains of action potentials during in vitro whole-cell recordings[21] we cannot exclude inactivation of voltage-gated ion channels *in*

vivo. Moreover, disinhibition (SOM-disinh) can be caused by SOMI-mediated silencing of presynaptic SOMIs or other types of GABAergic cells [95]. Note, that SOMI recruitment was defined as instantaneous and reliable action potential generation upon light onset whereas disinhibited units were characterized by a delayed recruitment with ≥ 5 ms after light onset (red striped line).

c Most SOMIs were persistently active during blue light delivery (n=92 cells, 18 mice, 21 recording sessions) whereas some showed light-triggered activity within the initial 5 ms time window after light onset that subsequently decreased during the light-on epoch. These units are identified as SOMIs inhibited by presynaptic SOMIs (n=46 cells, 16 mice, 19 recordings), indicating mutual connection among DG SOMIs[95].

d Some units showed marked increases in their discharges with >5 ms delay from light-onset and therefore defined as units disinhibited by SOMIs (SOM-disinh, n=75 cells, 18 mice, 22 recording sessions).

e Discharges of the majority of units were suppressed during blue light delivery and therefore defined as units inhibited by SOMIs (SOM-inh, n=389 units, 23 mice, 38 recording sessions).

f Pie charts summarize the fraction of the various types of light responsive cells and SOMI subtypes. Note 6.9% of all recorded units were identified as SOMIs.

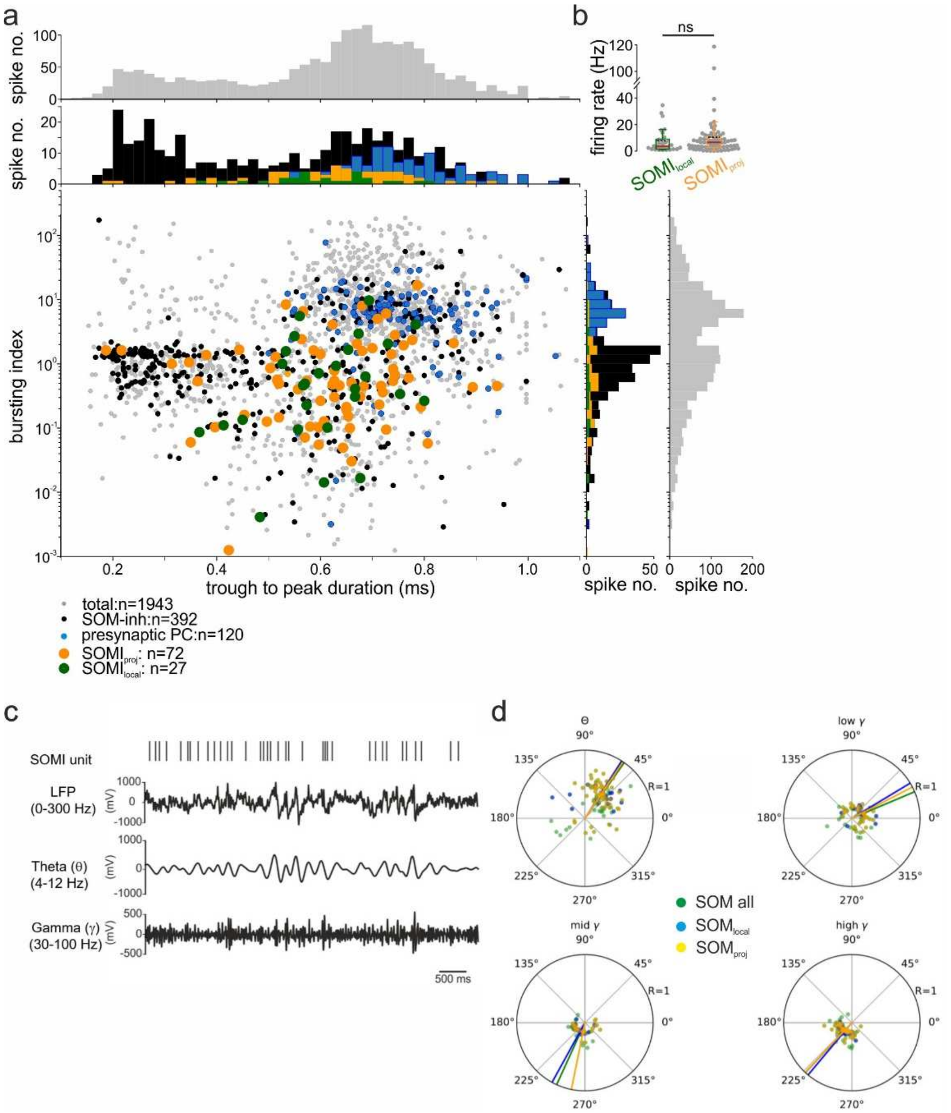

Supplementary Fig. 3. Single unit kinetics of SOMI subtypes.

a Unit classification based on optogenetic identification, trough-to-peak duration, bursting index and monosynaptic connections. Each dot corresponds to one unit (1943 units). Orange dots indicate $SOMI_{proj}$ while green dots represent $SOMI_{local}$. Black dots represent units inhibited by presynaptic SOMIs (SOM-inh). Putative presynaptic PCs identified by monosynaptic connections between cell pairs are shown in blue. Histograms show the distribution through-to-peak duration (top) and bursting index (right) for the entire recorded unit population (gray bars) and identified cell types (colored bars).

b Average firing rates of $SOMI_{local}$ and $SOMI_{proj}$ ($SOMI_{local}$ n=27 cells, $SOMI_{proj}$ n=72 cells; two-tailed unpaired t-test, P = 0.46).

c Timing of an optogentically identified SOMI unit in relation to the local field potential (LFP) in the hilar DG filtered with different band-pass filters.

d Circular plots depict SOMI subtype clusters during theta (upper left), low gamma (30-50 Hz, upper right), mid gamma (50-70 Hz, lower left) and high gamma (70-100 Hz, lower right) network oscillations showing their preferred angles to the corresponding oscillatory cycles. Colored lines relate to the mean cluster angles defined by the SOMI subtypes. 'All SOMIs' refers to cells, which could not be unequivocally identified as local vs projecting SOMIs. There is a lacking significant difference in the preferred average angle between $SOMI_{loc}$ and $SOMI_{proj}$ (two-tailed Kuiper tests for circular distributed variables taking into account only significantly modulated cells in the respective frequency band: theta P = 1.0 SOMI-local n=24, SOMI-proj n=64; low gamma P = 1.0, SOMI-local n=14, SOMI-proj n=35; mid gamma P = 1.0, SOMI-local n=14, SOMI-proj n=27; high gamma P = 1.0, SOMI-local n=15, SOMI-proj n=48). Circles represent individual cells. Bars in (b) with lines represent boxplots (boxes represent 25th to 75th percentiles; red line, median; black dashed line, mean; whiskers, the largest/smallest data point within the ± 1.5 IQR range); ns, not significant.

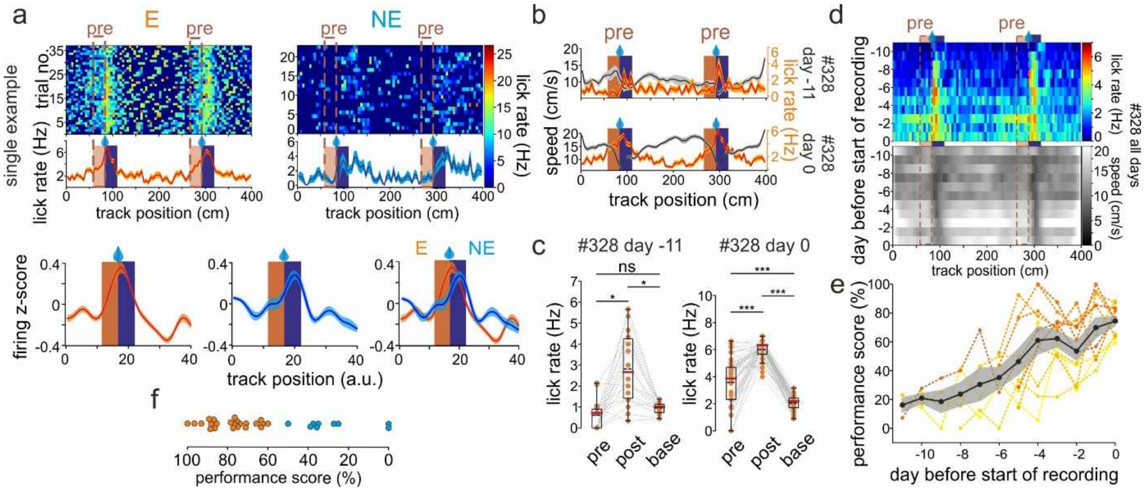

Supplementary Fig. 4. Behavioral analysis of mice performing a goal-oriented learning task.

a Top, single example of licking behavior of one E (left) and NE (right) mouse. Top, licks on subsequent laps plotted against track position with the rewards delivered at locations indicated by the symbol. Data were averaged over bins of 100 ms (single example of an E and NE animal). Below, average SOMI activity (z-scored; continuous line) across track from all animals (n=11 E and 6 NE mice). Line with shadow represents mean ± SEM z-scored firing rate. Brown and purple areas represent pre- and post-reward zones, respectively. Right, superposition of the traces shown on the left. Note increased SOMI discharge rate in the pre-reward zone in E animals.

b-d Goal-related reward learning in an example animal during progressive training days.

b Running speed (black) and lick rate (orange) are plotted across track position for one animal for training day -11 (upper traces) and day 0 (lower traces). Day 0 indicates the day where recording was started. On day -11 the animal did neither show a reduced mean running speed nor anticipatory licking in the pre-reward area (orange area). In contrast, running speed markedly declined and pre-reward licking increased on day 0.

c Quantification of mean lick rates in the pre-reward, post-reward area and the residual track (base) on day -11 and day 0 in E mice for same example as shown in (b) (two-tailed Friedman test; day -11: $P = 8.0 * 10^{-6}$; day 0: $P = 4.75 * 10^{-26}$).

d Summary of licking behavior (top) and running speed (bottom) during progressive training days obtained from one representative animal.

e Performance score defined as percentage of laps where increased lick rate was obtained in the pre-reward area compared to the remaining track for E mice, measured on subsequent training days. Black line with shadow represents mean ± SEM performance. Coloured circles connected by lines represent data from individual animals (9 mice). Circles connected by lines in c represent mean values from individual recording laps. *P < 0.05; **P < 0.01; ***P < 0.001; ns, not significant. Boxes represent 25th to 75th percentiles; red line, median; black dashed line, mean; whiskers, the largest/smallest data point within the ± 1.5 IQR range.

f Performance score remained higher in E (orange circles) as compared to NE mice (blue circles).

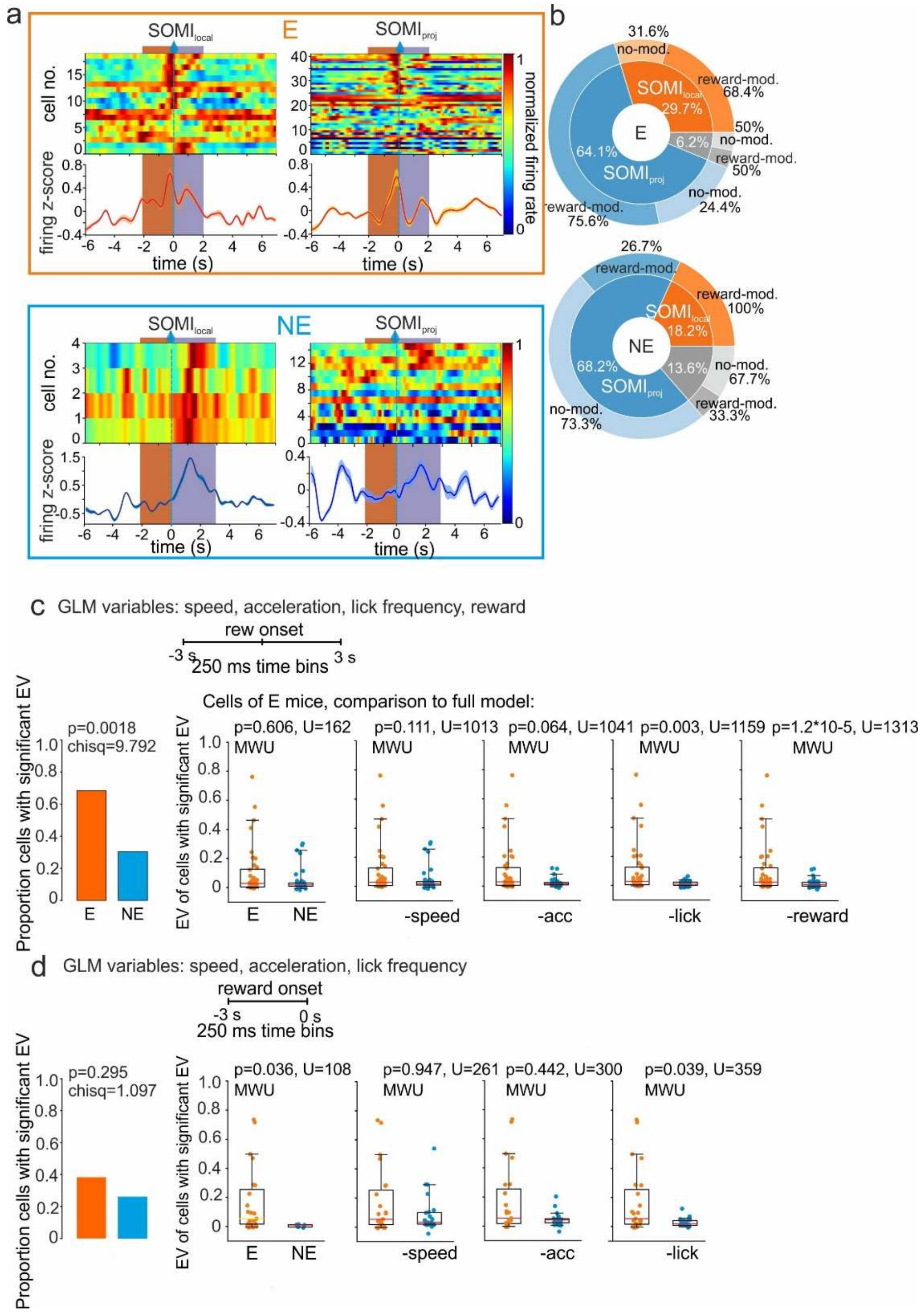

Supplementary Fig. 5. $\text{SOMI}_{\text{local}}$ and $\text{SOMI}_{\text{proj}}$ show similar expectation-related activity patterns at pre-reward sites.

a Summary of peak-normalized firing rates of all SOMIs obtained from E (top panel, orange) and NE (bottom panel, blue) mice plotted against time (bin size 100 ms). Top in each panel, individual SOMIs. Bottom in each panel, mean firing (z-scored) of all recorded SOMIs in E and NE mice (continuous line).

b Fractions of reward modulates $\text{SOMI}_{\text{local}}$ and $\text{SOMI}_{\text{proj}}$ in E (upper) and NE (lower) animals.

c From left to right, proportion of SOMIs with significant explained variance (EV) of spike rates at reward zones for E and NE mice using a generalized linear model (GLM) with behavioral predictors including lick rate, running speed and reward location (Chi-square test, P = 0.0018, Chi-square 9.792); EV of

spike rate from SOMIs' with significant EV at reward sites defined by the time window from -3 s prior and 3 s after reward onset (0 s) for E and NE mice using a GLM with all behavioural variables included or one variable removed. From left to right and bottom, without running speed (two-tailed Wilcoxon rank-sum test, P = 0.03), without lick rate (P = 0.56) or without reward location (P = 0.20). Bars with lines represent boxplots (boxes represent 25th to 75th percentiles; red line, median; black dashed line, mean; whiskers, the largest/smallest data point within the ± 1.5 IQR range). *P < 0.05; ns, not significant. Line with shadow represents mean ± SEM. Circles represent individual cells.

d Same as (c) for a time window from -3 s prior to reward onset, to immediately before reward onset (0 s). Reward can therefore not be used as a predictor in this GLM. For details see Methods and Source data extended Figures.

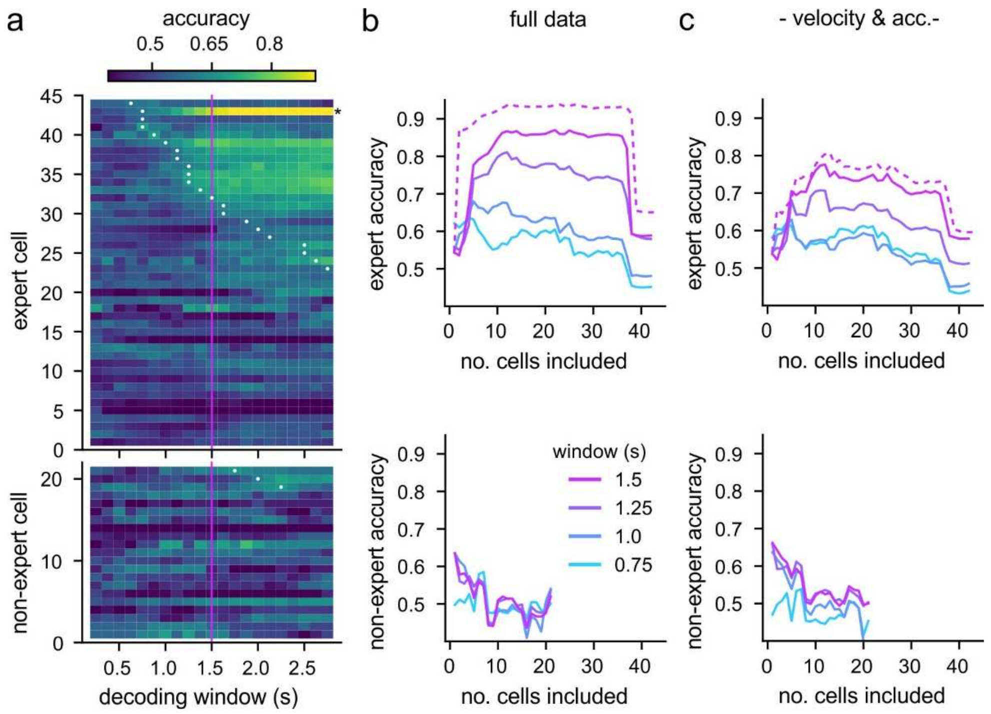

Supplementary Fig. 6: A small number of SOMIs sufficiently predict reward only in expert animals.

a Fraction of correct decoding (accuracy) from firing activity differences between baseline and the reward expectation period (1.5 s window prior to reward delivery as indicated by the purple line, up to 3 s in total) is shown for all SOMIs from expert (top) and non-experts (bottom) animals as a function of the decoding window length. Reward is administered 1.5 s (vertical purple line) after the start of the expectation period. White dots indicate the smallest window length for which decoding from a single SOMI was significantly larger than chance (alpha = 0.01). About one third of expert SOMIs allow significant reward prediction already on a single-cell level.

b Cross-validated population decoding accuracy as a function of the number of cells included in the probabilistic estimate for expert (top) and non-expert (bottom) animals. Cells were sorted according to

panel A from top to bottom. Colours encode different lengths of decoding windows (0.75 to 1.5 s). For window length 1.5 s we show both results, including the highly discharging cell (star in a, dashed bright purple) and excluding it (solid bright purple). For window length <1.5 s we excluded the highly discharging cell. Top, only ~10 SOMIs are necessary to predict the reward in experts with a success rate of 80% for a decoding window 1.5 s prior to reward onset. Bottom, the 80% criterion is never reached for non-experts.

c Same as (b) but spike counts are modified by subtracting estimates of speed and acceleration contributions according to the GLM regression. For details see Methods and Source data extended Figures.

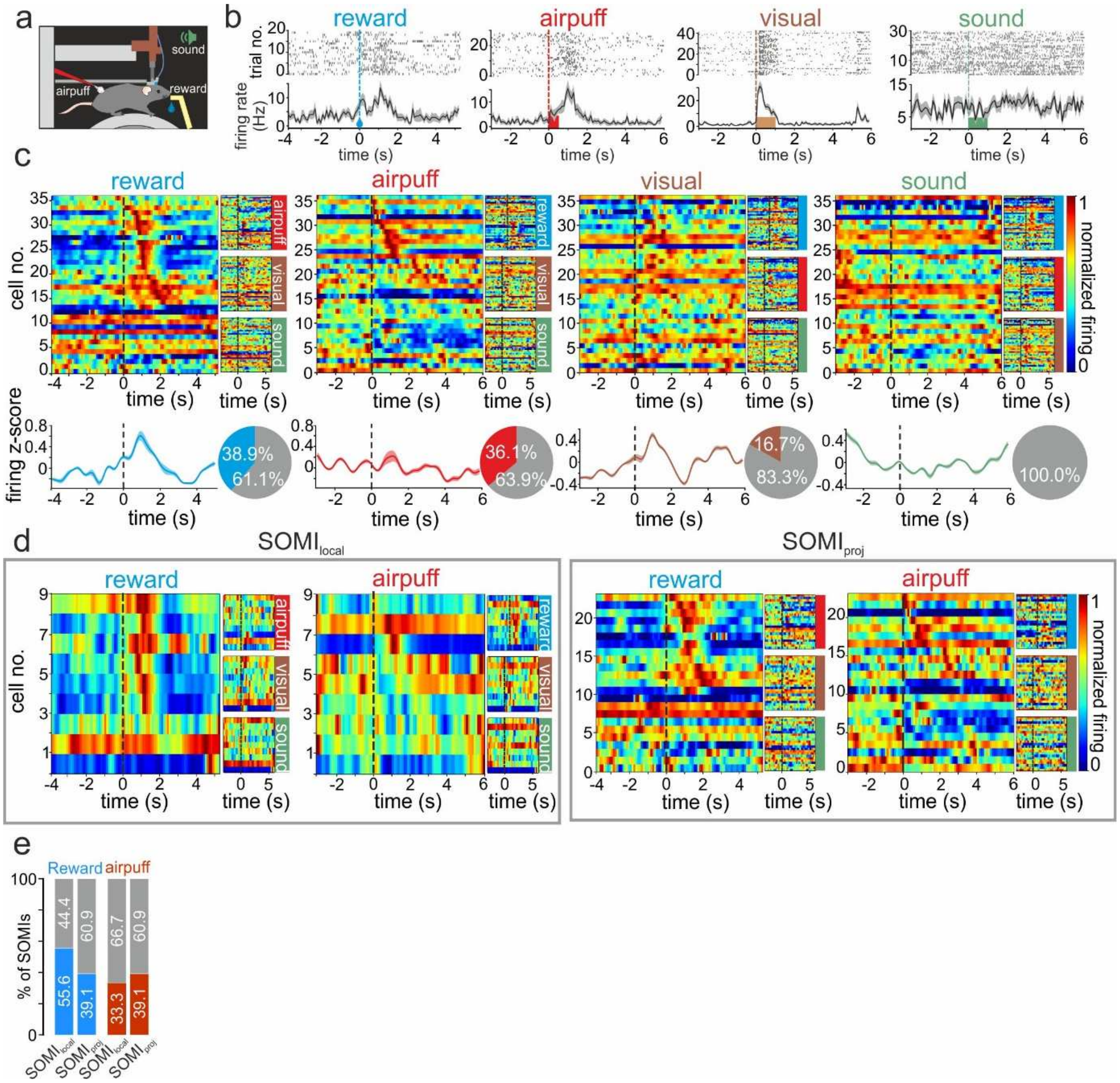

Supplementary Fig. 7. Multiple sensory-evoked responses by DG-SOMIs.

a Schematic illustration of diverse sensory modalities applied to the animal.

b Representative traces from individual SOMIs during the pseudo-random presentations of discrete sensory stimuli (reward, air-puff, visual and sound stimulation, see Methods for details). Vertical striped lines refer to time points of stimulation onset. Upper, individual trials; below, mean discharge frequency ± SEM. Black dots represent individual spikes from an example unit. Note that the activity rise during reward delivery was most likely not evoked by the sound of the reward pump (Methods).

c Upper, SOMI responses to distinct sensory stimuli ordered by the stimulation (n=36 cells, 6 mice). Bottom, lines with shadows represent average activity of SOMIs ± SEM; circular plots refer to the fraction of SOMIs responsive to individual stimulation modalities. A significant change in activity was defined by comparing the discharges of individual traces from each cell 3 s before and after stimulus onset at $P < 0.05$.

d The same as (c) for $SOMI_{local}$ (n=9 cells, 2 mice) and $SOMI_{proj}$ (n=23 cells, 6 mice) responding to reward and air-puff stimuli.

e Fractions of $SOMI_{local}$ and $SOMI_{proj}$ showing significant increase in activity after reward- and air-puff-mediated afferent stimulation (blue and orange). Gray areas depict fraction of cells, which did not respond to the stimuli.

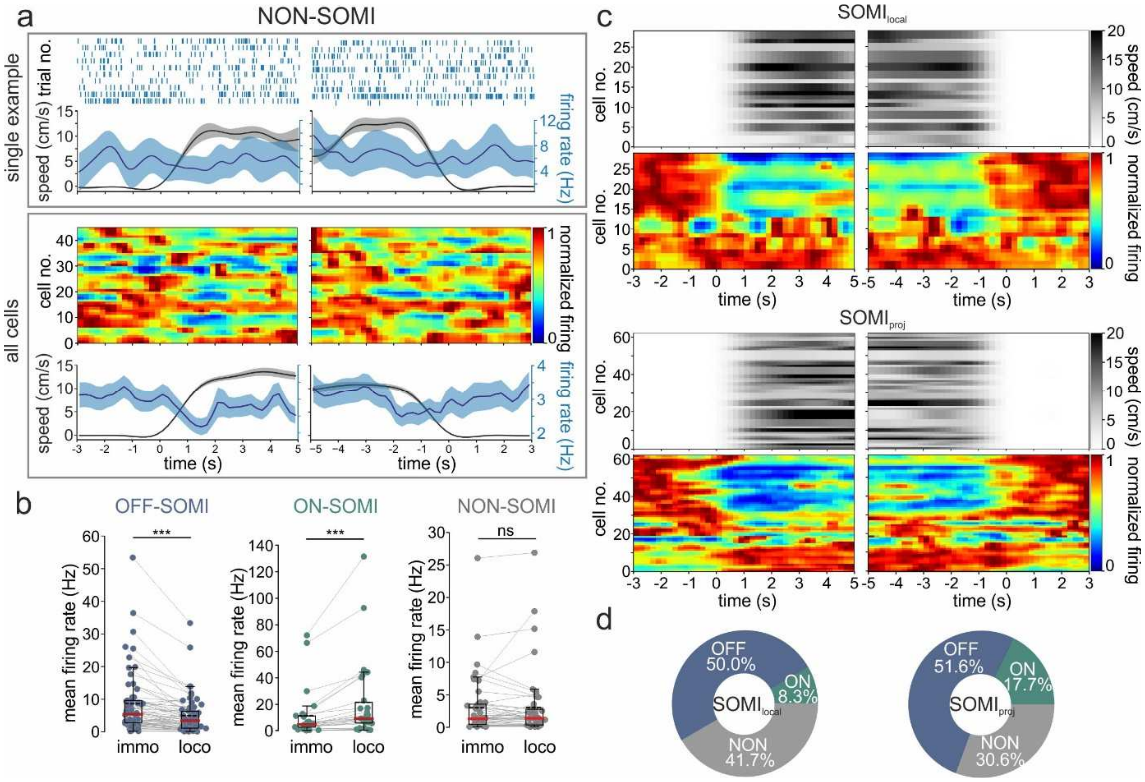

Supplementary Fig. 8. Both $SOMI_{local}$ and $SOMI_{proj}$ show ON- and OFF-SOMI properties.

a NON-SOMI activity during transitions from immobility to locomotion. Upper square depicts activity of a single NON-SOMI aligned to running on- (left) and offsets (right). Bottom square depicts activities of all optogenetically-identified NON-SOMIs normalized to their maximal firing in relation to running on- and offsets (n=45 cells, 28 mice). Note that these cells do not show unidirectional activity changes during transitions between immobility and locomotion.

b Mean firing rates of OFF-, ON- and NON-SOMIs during immobility (immo) and locomotion (loco). Bars with lines represent boxplots (boxes represent 25th to 75th percentiles; red line, median; black dashed line, mean; whiskers, the largest/smallest data point within the ± 1.5 IQR range). Each filled circle represents a cell (n=61 OFF-SOMIs, two-tailed Wilcoxon sign-rank test, $P = 8.30*10^{-6}$; n=26 ON-SOMIs, $P = 1.75*10^{-8}$; n=45 NON-SOMIs, P = 0.41).

c Activity of $SOMI_{local}$ and $SOMI_{proj}$ during transitions between immobility and locomotion of OFF-, ON- and NON-SOMIs.

d Fractions of $SOMI_{local}$ or $SOMI_{proj}$ with OFF-, ON- and NON-SOMI discharge characteristics. Lines with shadow represent mean ± SEM. Circles connected by lines represent individual cells. *** P < 0.001; ns, not significant.

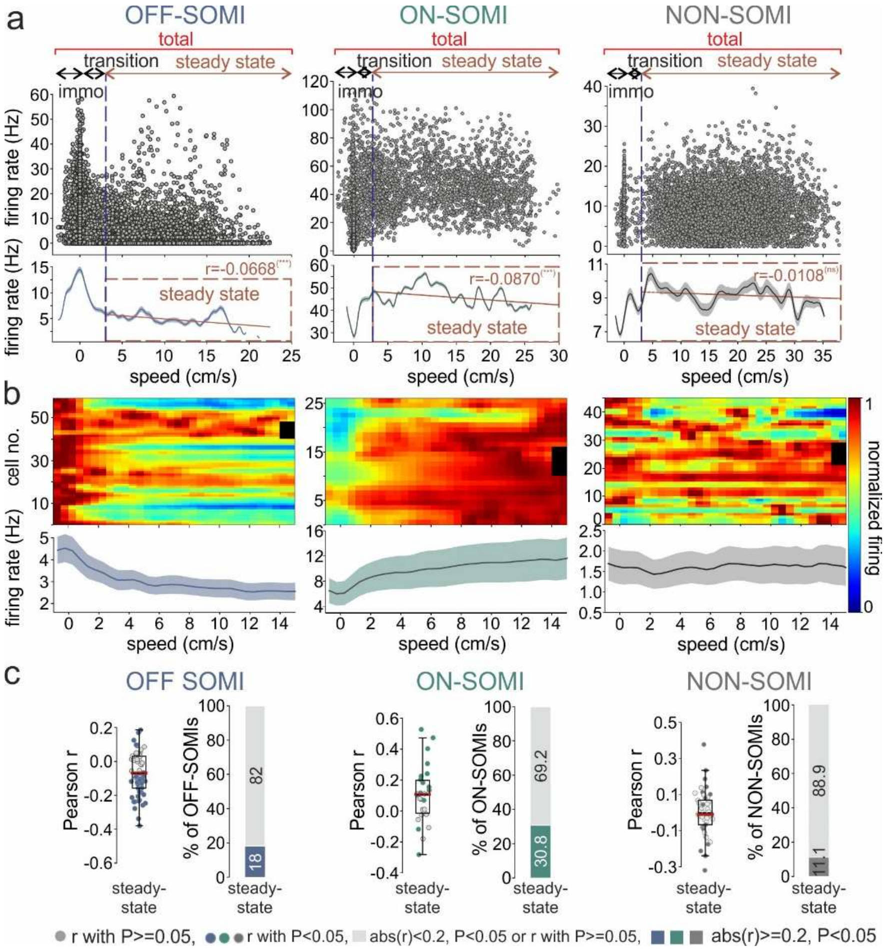

Supplementary Fig. 9. The activity of the majority of OFF- and ON-SOMIs is not steady-state speed modulated.

a Activity of a single OFF- (left), ON- (middle) and NON-SOMI (right) is plotted against running speed. Top, each circle represents the firing rate plotted against the running speed of an example cell averaged over time bins of 100 ms. Bottom, line with shadow shows mean activity ± SEM for the same cell. Vertical striped line represent the transition between immobility and steady-state locomotion (≥ 3 cm s^{-1}). Data from each cell were fit to a least-square linear regression for assessing correlation between cell firing and animal's velocity as defined by the Pearson r.

b Top, heat-plot shows peak-normalized activity of all identified OFF-, ON- and NON-SOMIs as a function of running speed (0.2 cm s^{-1} bins). Bottom line with shadows represents mean ± SEM discharge rates as a function of running speed.

c Left bars in each of the three panels are summary plots showing individual Pearson correlation coefficients (circles represent individual cells) calculated during the steady-state running epochs as defined in (a) for OFF- (blue), ON- (green) and NON-SOMIs (gray); boxes representing 25th to 75th percentiles; red line indicate the median, black dashed line the mean and whiskers, the largest/smallest data point within the ± 1.5 IQR range. Right bars in each of the three panels show the fraction of SOMIs with no significant steady-state speed modulation (gray areas) and the small fraction of SOMIs that are modulated during steady-state running periods (coloured areas; Pearson r > 0.2 or < -0.2). Note that the majority of OFF-, ON- and NON-SOMIs do not show significant correlation between their activity and

steady-state running speed (n=61 OFF-SOMI; n=26 ON-SOMIs; n=45 NON-SOMIs in 28 mice; OFF-cells: 82%, Pearson's r ≥ -0.2; ON-cells: 69.2%, Pearson's r ≤ 0.2; NON: 89%, r ≤ 0.2).

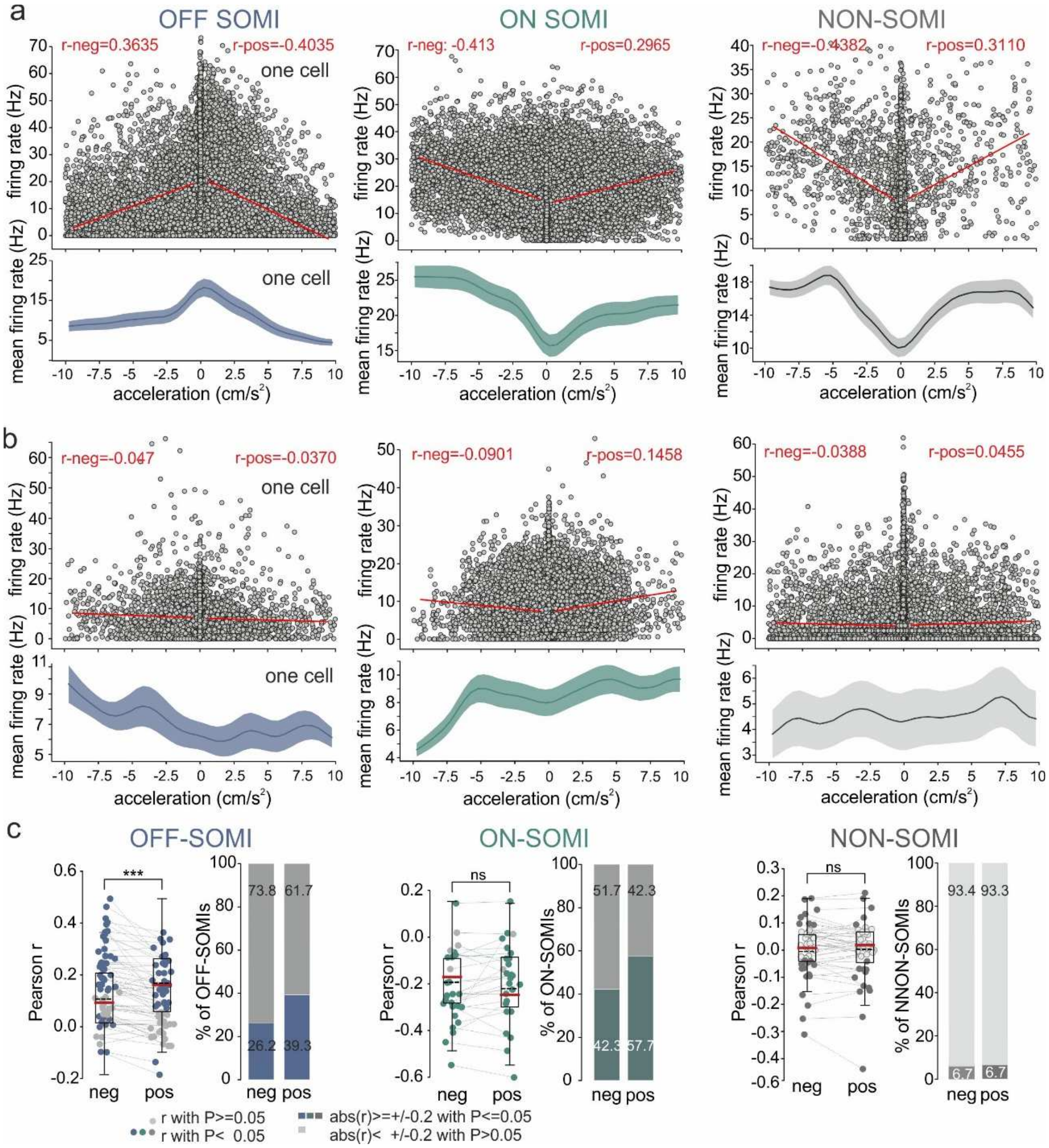

Supplementary Fig. 10: The activity of the majority of OFF- and ON-SOMIs is not modulated by acceleration.

a,b Activity of a single OFF- (left), ON- (middle) and NON-SOMI (right) is plotted against acceleration during running. *Top*, each circle represents the firing rate plotted against acceleration of an example cell averaged across time bins of 100 ms. Data from each cell were fit to a least-square linear regression for assessing correlation between cell firing and animal's acceleration as defined by the Pearson r. Examples in (a) show cells with significant changes in their activity related to acceleration whereas in (b) cells without significant acceleration-dependent activity changes.

c Left bars in each of the three panels are summary plots showing individual Pearson correlation coefficients (circles represent individual cells) as defined in (a) for OFF- (purple), ON- (green) and NON-SOMIs (gray); boxes representing 25th to 75th percentiles; red line indicate the median, black dashed line the mean and whiskers, the largest/smallest data point within the ± 1.5 IQR range. Right bars in each of the three panels show the fraction of SOMIs with no significant acceleration modulation (gray areas) and the fraction of SOMIs that are modulated by acceleration (coloured areas; Pearson r > 0.2 or < -0.2). Note that the majority of OFF-, ON- and NON-SOMIs do not show significant correlation between their activity and acceleration (n=61 OFF-SOMI; n=26 ON-SOMIs; n=45 NON-SOMIs in 28 mice; OFF-cells: 73.8% negative and 61.7% positive, Pearson's r ≥ -0.2 or 0.2; ON-cells: 51.7.2% positive and 42.4% negative modulated, Pearson's r ≤ 0.2 or -0.2; NON: 93.4% positive and 93.3% negative, r ≤ 0.2 or -0.2). *** P < 0.005; ns not significant. OFF neg vs pos P = 1.7422 10^{-11}, two-tailed paired t-Test; ON pos vs neg P = 0.05206, two-tailed paired t-test; NON pos vs neg P = 0.509, two-tailed Wilcoxon test.

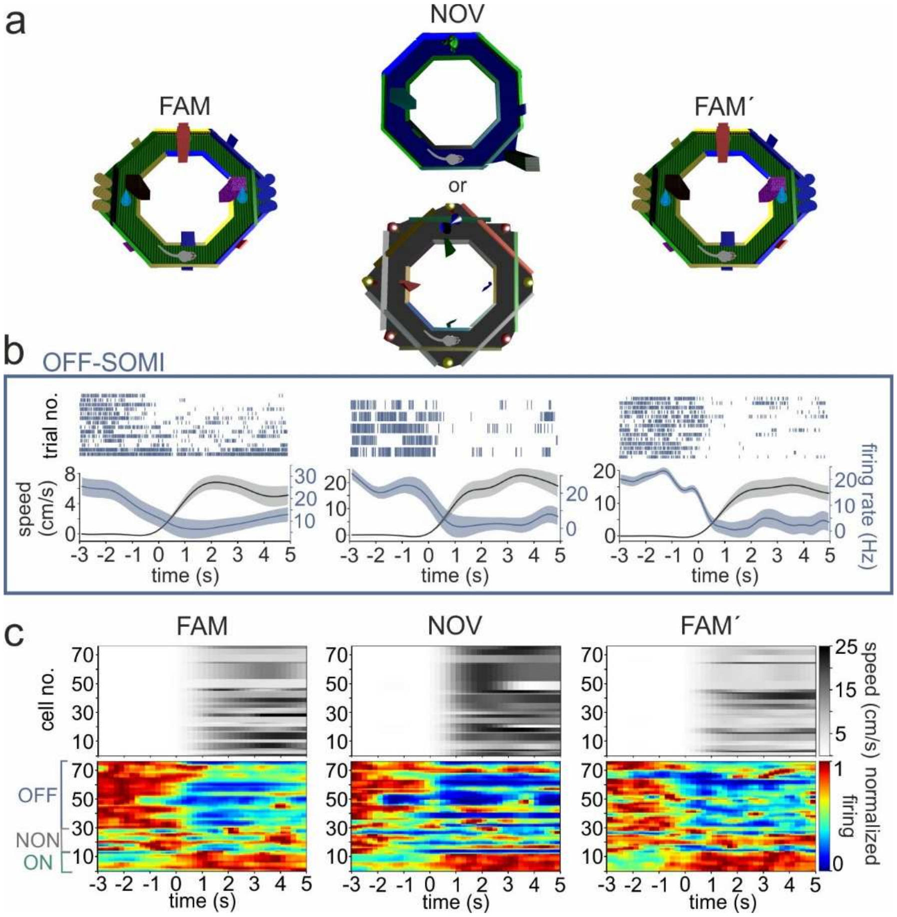

Supplementary Fig. 11. Characteristics of ON- and OFF-SOMI activity is independent of context and sessions.

a Schematic illustration of the familiar (FAM) and a novel (NOV) virtual environment. As animals were recorded on two consecutive days, we used two distinct novel environments for the same animal with each presented on one of the two days to ensure novelty of the environment to the animal.

b Representative OFF-SOMI recordings during transitions between immobility and locomotion in the FAM (left) and NOV (middle) environment and after re-exposure to the original familiar (FAM’, right) context. Note that activity changes of example OFF-SOMIs, remain stable across contexts (FAM vs NOV) and across sessions (FAM vs FAM’).

c Summary of OFF-, ON- and NON-SOMI activities during transitions between immobility and locomotion in the FAM, NOV and FAM’ context. Top, mean speed changes during running onsets for recorded cells. Bottom, heat plot represents peak-normalized cell activities aligned to transitions between immobility and locomotion for OFF-, ON- and NON-SOMIs. Lines with shadows represent mean ± SEM.

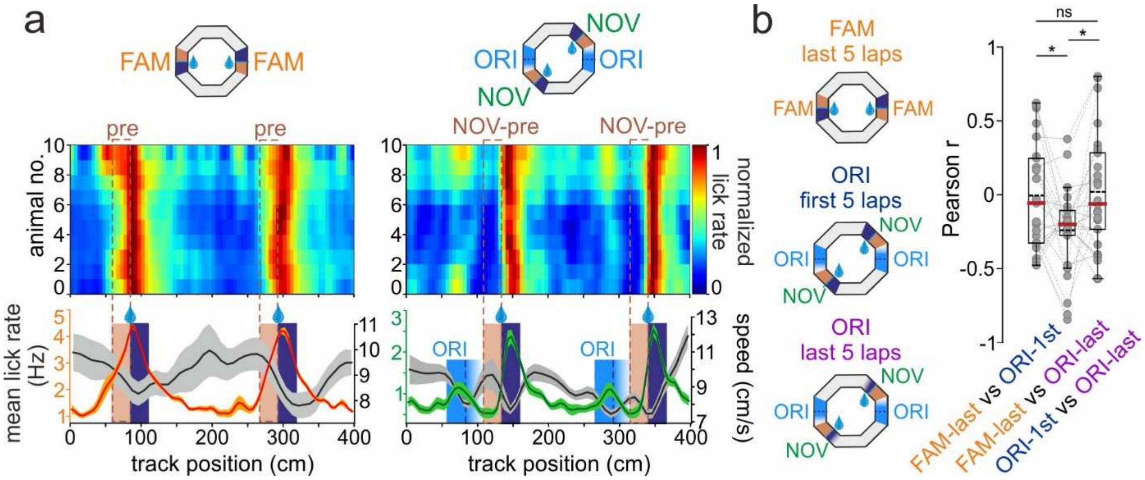

Supplementary Fig. 12. Quantification of lick behavior and SOMI activity during the transition from the familiar to a novel previously unrewarded zone and the now non-rewarded original reward zone.

a Top, schematic illustration of the reward-translocation experiment. Middle, lick-rate normalized to its maximal value was plotted for each animal as a function of track position in the familiar context (FAM, left) and the novel (NOV) reward location (right) as well as in the original familiarized reward site (ORI) after reward translocation (n=10 mice). Below, the same but averaged across all animals. Note anticipatory licking in the pre-reward zone was lost after translocation in the NOV reward site (right, green trace) and remained at lower values in the original pre-reward area (blue area, green trace). Running speed declined in the pre-reward FAM area but not in the pre-reward NOV region.

b Left, schematic illustrations of the laps in the FAM and ORI area, which have been selected for the comparison shown on the right. Right, summary plot showing the comparison of SOMI activity in the post-reward area between the last 5 laps in the FAM context and the first and last 5 laps after reward translocation in the ORI area. Note that the correlation coefficients between the last 5 laps in the FAM and the 1st 5 laps after translocation in the ORI post-reward zone are higher than those between the last 5 laps in the FAM context and the last 5 out of 35 laps in the ORI post-reward zone after translocation (two-tailed Friedman test, $P = 0.039$), indicating that mice rapidly learned across laps that rewards are no longer available at the ORI zone. Circles represent individual cells (n=27). Lines with shadows represent mean ± SEM. Boxes represent 25th to 75th percentiles; red line, median; black dashed line, average; whiskers, the largest/smallest data point within the ± 1.5 IQR range; $^{*}P < 0.05$; ns, not significant (two-tailed paired t-Test).

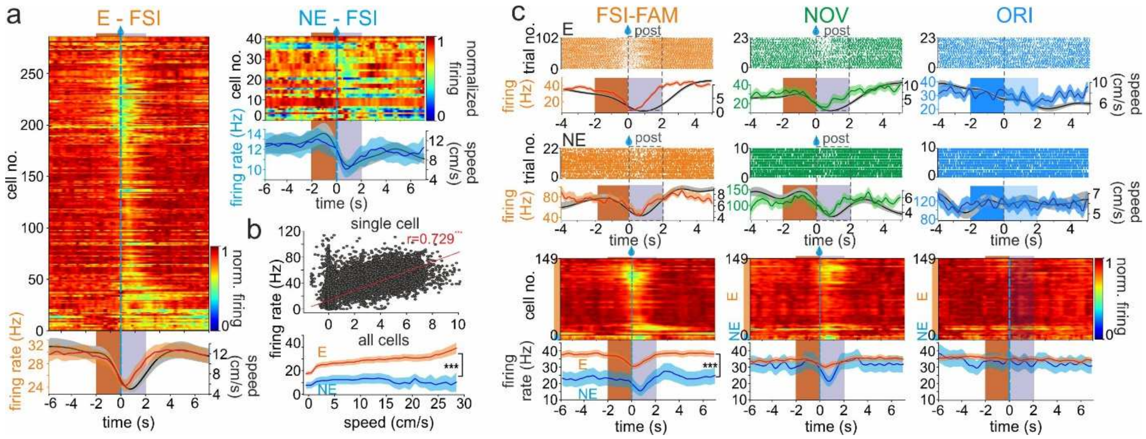

Supplementary Fig. 13: Fast-spiking interneurons (FSIs) do not show predictive goal coding in experts.

a Activity of all FSIs near the reward zone in E and NE mice (E, n=285 cells, 11 mice; NE, n=42 cells, 6 mice). Top, firing of each FSI is normalized to its peak activity. Bottom, average of z-scored FSI activity is superimposed with the average running speed (black) and aligned to reward onset. Brown square indicates pre-reward and purple square the post-reward area.

b Upper, firing rate of a single FSI plotted against time. Data were fit to a least-square linear regression for assessing correlation between cell firing and animal's speed as defined by the Pearson r. Below, lines represents the mean activity from all FSIs obtained from expert (E) and non-expert (NE) mice ($P=1.7343\ 10^{-6}$, two-tailed Wilcoxon sign-rank),

c Upper two rows, Raster plots of a single FSI obtained from an E and NE animal (upper two rows) in familiar (FAM), novel (NOV) and original trained reward areas (ORI). Dots represent individual action potentials. Coloured lines represent average activity of a single FSI across all trials and are superimposed with the average running speed (black line). Lower two rows, same as upper two rows but for all recorded FSIs in E and NE mice (orange vs blue line). Lines with shadows represent mean ± SEM; dots in b represent individual spikes; *** P < 0.005.
