# Supplementary material for: Predictive goal coding by dentate gyrus somatostatin-expressing interneurons in male mice
Source: Nat Commun. 2025 Jun 25;16:5382. doi: 10.1038/s41467-025-60841-y (PMC12198415; doi:10.1038/s41467-025-60841-y)
Supplement: Supplementary file 2 — Reporting summary [file 41467_2025_60841_MOESM2_ESM.pdf]

nature portfolio

Corresponding author(s): Marlene Bartos

Last updated by author(s): May 13, 2025

Reporting Summary

Nature Portfolio wishes to improve the reproducibility of the work that we publish. This form provides structure for consistency and transparency in reporting. For further information on Nature Portfolio policies, see our Editorial Policies and the Editorial Policy Checklist.

Statistics

For all statistical analyses, confirm that the following items are present in the figure legend, table legend, main text, or Methods section.

n/a	Confirmed	
☐	☒	The exact sample size (*n*) for each experimental group/condition, given as a discrete number and unit of measurement
☐	☒	A statement on whether measurements were taken from distinct samples or whether the same sample was measured repeatedly
☐	☒	The statistical test(s) used AND whether they are one- or two-sided<br>*Only common tests should be described solely by name; describe more complex techniques in the Methods section.*
☒	☐	A description of all covariates tested
☐	☒	A description of any assumptions or corrections, such as tests of normality and adjustment for multiple comparisons
☐	☒	A full description of the statistical parameters including central tendency (e.g. means) or other basic estimates (e.g. regression coefficient) AND variation (e.g. standard deviation) or associated estimates of uncertainty (e.g. confidence intervals)
☐	☒	For null hypothesis testing, the test statistic (e.g. *F*, *t*, *r*) with confidence intervals, effect sizes, degrees of freedom and *P* value noted<br>*Give P values as exact values whenever suitable.*
☒	☐	For Bayesian analysis, information on the choice of priors and Markov chain Monte Carlo settings
☒	☐	For hierarchical and complex designs, identification of the appropriate level for tests and full reporting of outcomes
☐	☒	Estimates of effect sizes (e.g. Cohen's *d*, Pearson's *r*), indicating how they were calculated

Our web collection on statistics for biologists contains articles on many of the points above.

Software and code

Policy information about availability of computer code

Data collection	Single unit recordings with Python3.7.11; custom analysis routines written in Python3.7.
Data analysis	Python3.7.11; custom analysis routines written in Python3.7, decoder using are deposited in a public repository (GitHub; https://github.com/cleibold/somireward).

For manuscripts utilizing custom algorithms or software that are central to the research but not yet described in published literature, software must be made available to editors and reviewers. We strongly encourage code deposition in a community repository (e.g. GitHub). See the Nature Portfolio guidelines for submitting code & software for further information.

Data

Policy information about availability of data

All manuscripts must include a data availability statement. This statement should provide the following information, where applicable:

- Accession codes, unique identifiers, or web links for publicly available datasets
- A description of any restrictions on data availability
- For clinical datasets or third party data, please ensure that the statement adheres to our policy

All data are available in the paper and Extended Data. The full data set used in this study (single units, spike times, phase relationship) are uploaded with the manuscript. Identifiers are provided.

Research involving human participants, their data, or biological material

Policy information about studies with human participants or human data. See also policy information about sex, gender (identity/presentation), and sexual orientation and race, ethnicity and racism.

Reporting on sex and gender	No human participants were included in this study.
Reporting on race, ethnicity, or other socially relevant groupings	No human participants were included in this study.
Population characteristics	No human participants were included in this study.
Recruitment	No human participants were included in this study.
Ethics oversight	No human participants were included in this study.

Note that full information on the approval of the study protocol must also be provided in the manuscript.

Field-specific reporting

Please select the one below that is the best fit for your research. If you are not sure, read the appropriate sections before making your selection.

☒ Life sciences ☐ Behavioural & social sciences ☐ Ecological, evolutionary & environmental sciences

For a reference copy of the document with all sections, see nature.com/documents/nr-reporting-summary-flat.pdf

Life sciences study design

All studies must disclose on these points even when the disclosure is negative.

Sample size	No statistical methods were used to determine sample size prior to experimentation. Sample sizes are similar to previous publications.
Data exclusions	Animals were only excluded if the location of the silicon probe did not reach the hilar dentate gyrus. Otherwise no animals were excluded.
Replication	Findings of Fig. 1, 2, 3, 4 were performed as three independent replications with groups of 5-7 mice each. Data shown in Figure 5 were repeated in 2 independent replications with 4 mice in controls and 5 mice for hM4Di-mediated SOMI silencing in total.
Randomization	Randomization was not applied as comparisons were performed within the same animals.
Blinding	Blinding was not applied as comparisons were performed within the same animals.

Reporting for specific materials, systems and methods

We require information from authors about some types of materials, experimental systems and methods used in many studies. Here, indicate whether each material, system or method listed is relevant to your study. If you are not sure if a list item applies to your research, read the appropriate section before selecting a response.

Materials & experimental systems

n/a	Involved in the study	
☐	☒	Antibodies
☒	☐	Eukaryotic cell lines
☒	☐	Palaeontology and archaeology
☐	☒	Animals and other organisms
☒	☐	Clinical data
☒	☐	Dual use research of concern
☒	☐	Plants

Methods

n/a	Involved in the study	
☒	☐	ChIP-seq
☒	☐	Flow cytometry
☒	☐	MRI-based neuroimaging

Antibodies

Antibodies used	We used anti-SOM antibodies (1:500, rabbit, Peninsula Laboratories LLC. LO A18PO21141; cat. No. T-4102; specificity was tested with a competitive ELISA) and secondary antibodies: rabbit-Cy3 (1:1000, rabbit, Jackson ImmunoResearch, UK; LOT 130329; cat. no. 111-165-003; specificity tested with immunoelectrophoresis and ELISA).

Validation	This primary antibody has been tested and validated in ELISA against Somatostatin-14, the secondary antibody tested and validated with immunoelectrophoresis and ELISA.

Animals and other research organisms

Policy information about studies involving animals; ARRIVE guidelines recommended for reporting animal research, and Sex and Gender in Research

Laboratory animals	Adult male B6J.Cg-Ssttm 2.1(cre)Zjh/MwarJ and Ssttm2.1(cre)Zjh/J mice (SOM-Cre, 6-20 postnatal week, Jackson Laboratory, Stock No: 028864, 013044) were used. Groups of 2-4 mice were housed / cage on a non-reversed 12-h light-dark cycle (procedures were conducted during light phases).
Wild animals	No wild animals were used in this study.
Reporting on sex	Male mice were used in this study to minimize potential variability.
Field-collected samples	No field-collected samples are included in this study.
Ethics oversight	Regierungspräsidium Freiburg, license number G19/035 and G21/127.

Note that full information on the approval of the study protocol must also be provided in the manuscript.

Plants

Seed stocks	NA
Novel plant genotypes	NA
Authentication	NA
